# Comparative Analysis of Fully Guided and Free-Hand Orthognathic Surgery: Advancements, Precision, and Clinical Outcomes

**DOI:** 10.3390/dj13060260

**Published:** 2025-06-11

**Authors:** Sophia Tsokkou, Ioannis Konstantinidis, Antonios Keramas, Georgios Kiosis, Kanellos Skourtsidis, Danai Alexiou, Georgia-Nektaria Keskesiadou, Sofia Karachrysafi, Theodora Papamitsou, Ioannis Chatzistefanou

**Affiliations:** Research Team “Histologistas”, Interinstitutional Postgraduate Program “Health and Environmental Factors”, Department of Medicine, Faculty of Health Sciences, Aristotle University of Thessaloniki, 54124 Thessaloniki, Greece; ikonsc@auth.gr (I.K.); antonios@auth.gr (A.K.); gkiosis@auth.gr (G.K.); kskour@auth.gr (K.S.); dalexc@auth.gr (D.A.); gwgwkeske@gmail.com (G.-N.K.); sofia_karachrysafi@outlook.com (S.K.); thpapami@auth.gr (T.P.)

**Keywords:** full-guided, free-hand, mandibular reconstruction, computer assistant surgery, virtual surgical planning (VSP), CAD/CAM, dynamic navigation

## Abstract

**Background/Objectives**: Orthognathic surgery has evolved with digital advancements, improving precision and predictability. Traditional free-hand techniques rely on surgeon expertise, often leading to variable outcomes. Fully guided approaches integrate computer-assisted surgery, including virtual surgical planning (VSP), CAD/CAM, and dynamic navigation, enhancing accuracy and efficiency. This review compares these approaches, assessing their impact on surgical accuracy, efficiency, and patient outcomes. **Methods**: A scoping review was conducted across PubMed, MEDLINE, Scopus, Cochrane Library, and Embase databases, focusing on clinical trials and cohort studies. Key parameters analyzed include surgical precision, operative efficiency, complication rates, and functional/aesthetic results. **Results**: Fully guided techniques achieve sub-millimetric accuracy with mean length deviations ranging from 1.3 mm to 2.4 mm and mean angular deviations between 2.29° and 3.51°. Moreover, these approaches markedly reduce operative time, averaging between 34 min and 1.7 h, and postoperative complications. Digital tools streamline workflow, improving reproducibility and aesthetic outcomes. Free-hand methods remain cost-effective but require greater surgical expertise, often resulting in longer recovery periods and higher variability. **Conclusions**: Computer-assisted orthognathic surgery enhances precision and efficiency, outperforming free-hand techniques in accuracy and predictability. While free-hand methods remain viable for simpler cases, fully guided approaches optimize surgical execution. Future research should explore hybrid strategies combining digital precision with manual adaptability to further refine surgical techniques.

## 1. Introduction

Orthognathic surgery has undergone a transformative evolution with the integration of digital technologies, significantly enhancing precision, predictability, and patient outcomes. Orthognathic procedures have been historically characterized by the use of free-hand surgical techniques that rely on the surgeon’s expertise, manual adjustments, and conventional occlusal wafers to achieve skeletal repositioning. However, these methodologies are inherently constrained by the variability of both intra- and inter-operators, which may result in postoperative discrepancies and prolonged hospital stays [1,2].

The invention of computer-assisted surgery has revolutionized the field even further by, incorporating artificial intelligence (AI), three-dimensional imaging, dynamic navigation, augmented reality, and real-time imaging to optimize surgical accuracy. These technologies facilitate comprehensive preoperative planning, allowing surgeons to analyze anatomical variations and mitigate risks associated with tissue damage. Virtual surgical planning (VSP), combined with computer-aided design/computer-aided manufacturing (CAD/CAM), enables the creation of patient-specific surgical guides and pre-bent osteosynthesis plates, streamlining intraoperative execution and improving functional and aesthetic outcomes [3,4,5].

Recent studies have demonstrated that intraoperative navigation significantly enhances surgical precision, particularly in vertical positioning, which remains a challenge with traditional techniques. Navigation systems consistently achieve accuracy within 2 mm, offering superior control in the cranial-caudal dimension compared to conventional occlusal wafers [6]. Additionally, augmented reality-assisted free-hand orthognathic surgery has emerged as a promising approach, utilizing electromagnetic tracking and skin-attached dynamic reference tools to improve intraoperative visualization and accuracy [2].

Regardless of the advantages provided via computer-assisted surgery, free-hand techniques remain relevant, particularly for simpler maxillofacial cases. While free-hand procedures are more cost-effective, they often result in longer hospital stays and less predictable postoperative outcomes. The hierarchy of surgical stability in orthognathic surgery suggests that procedures involving maxillary expansion and mandibular rotation present higher instability risks, reinforcing the need for advanced digital planning [7].

Given these innovative capabilities, the present scoping review aims to systematically explore and critically appraise the application of fully guided techniques compared to conventional free-hand methods in orthognathic surgery, evaluating their respective advantages, limitations, and clinical implications. By synthesizing current research on digital surgical planning, intraoperative navigation, and custom guide fabrication, this review seeks to highlight the transformative impact of digital technologies on surgical precision and patient outcomes.

## 2. Materials and Methods

A scoping review approach was adopted for this manuscript in lieu of a formal systematic review due to the heterogeneous nature of study designs, planning methodologies, execution strategies, and outcome measures encountered in the literature addressing fully guided versus free-hand techniques in orthognathic surgery. This methodological choice was made to comprehensively map the current body of research, capturing the variability in digital (fully guided) and conventional free-hand approaches without forcing disparate data into a single quantitative synthesis. By using a scoping review framework, the study aimed to delineate critical gaps in the literature regarding the comparative effectiveness, reproducibility, operative efficiency, and clinical outcomes between these two surgical methods. Furthermore, this approach facilitates a foundational understanding of implementation strategies across diverse clinical contexts and elucidates the practical challenges associated with integrating advanced digital technologies into routine orthognathic surgical practice. Ultimately, the findings are intended to inform future systematic reviews and guide translational research efforts in the evolving field of craniofacial surgery. A detailed protocol for this scoping review was systematically developed, and all results were documented following the PRISMA for Scoping Reviews (PRISMA-ScR) checklist adapted for scoping reviews, as detailed in Supplementary Table S1 [8].

### 2.1. Identifying Research Questions

To clarify the research query, a PICO (population, intervention, comparator, outcome) framework was employed, as detailed in Table 1. Accordingly, the following review question was developed:

“Among adult (≥18 years) patients undergoing orthognathic surgery, what is the evidence regarding the efficacy of fully guided (digital, computer-assisted) surgical approaches, incorporating virtual surgical planning, CAD/CAM, and 3D imaging, in improving surgical accuracy, operative efficiency, functional and aesthetic outcomes, and reducing complication rates, compared with conventional free-hand techniques, over the past twenty years?”.

### 2.2. Identifying Relevant Studies

A comprehensive search was conducted on 1 May 2025 across multiple biomedical databases, including PubMed, MEDLINE, Scopus, Cochrane Library and Embase, using the following query strings: ((“orthognathic surgical procedures”[MeSH Terms] OR (“orthognathic”[All Fields] AND “surgical”[All Fields] AND “procedures”[All Fields]) OR “orthognathic surgical procedures”[All Fields] OR (“orthognathic”[All Fields] AND “surgery”[All Fields]) OR “orthognathic surgery”[All Fields] OR “orthognathic surgery”[MeSH Terms] OR (“orthognathic surgical procedures”[MeSH Terms] OR (“orthognathic”[All Fields] AND “surgical”[All Fields] AND “procedures”[All Fields]) OR “orthognathic surgical procedures”[All Fields] OR (“jaw”[All Fields] AND “surgery”[All Fields]) OR “jaw surgery”[All Fields])) AND ((“free”[All Fields] AND (“hand”[MeSH Terms] OR “hand”[All Fields])) OR (“full”[All Fields] AND (“guide”[All Fields] OR “guided”[All Fields] OR “guides”[All Fields] OR “guiding”[All Fields])))) for PubMed, MEDLINE, Cochrane, and Embase and (TITLE-ABS-KEY (orthognathic AND surgery) OR TITLE-ABS-KEY (jaw AND surgery)) AND (TITLE-ABS-KEY (free AND hand) OR TITLE-ABS-KEY (full AND guided)) for Scopus. References were reviewed for identification of any additional relevant articles.

### 2.3. Study Selection—Eligibility and Screening

The review was restricted to full-text research studies published in peer-reviewed journals in the English language. Eligible studies had to meet the following criteria: the study must include adult (≥18 years) patients undergoing orthognathic surgery; the study design must be a clinical trial, prospective or retrospective cohort study, or case report; the study must primarily focus on comparing the effectiveness of fully guided (digital, computer-assisted) approaches versus conventional free-hand techniques in orthognathic surgery, assessing outcomes such as surgical accuracy, operative efficiency, functional and aesthetic results, and complication rates; and the study must be published from 2001 until 2025. Duplicate records, review articles, systematic reviews and meta-analyses, protocols and guidelines, animal studies, conference abstracts and presentations, preprints, clinical trials under patient recruitment or without published results, ongoing clinical trials, and studies deemed irrelevant were excluded.

After excluding articles according to the above criteria using automated tools and researcher screening, the final set of articles was retrieved. To ensure accuracy and objectivity, two independent reviewers (I.K. and S.T.) initially screened titles and abstracts in a double-blinded process. For studies that passed this initial screening, full texts were obtained and further evaluated to determine final eligibility. Any discrepancies during the screening process were resolved by a third reviewer (T.P.).

### 2.4. Data Charting

Key variables were systematically extracted from all included studies by the primary researchers (I.K. and S.T.). The extracted variables included the following: first author, publication year, study design, sample size, and patient demographic characteristics (mean age). In addition, specific details pertaining to the surgical intervention were charted, such as the type of orthognathic procedure performed (e.g., mandibular, maxillary, or bimaxillary surgery), the intervention type (fully guided digital approach employing virtual surgical planning [VSP], CAD/CAM, and 3D imaging versus conventional free-hand techniques), and details of the intraoperative and preoperative planning protocols used. Outcome measures were also recorded, including metrics of surgical accuracy (e.g., linear and angular deviations, occlusion alignment, and condyle positioning), operative parameters (total operative time, ischemia time, and preoperative planning time), complication rates, and functional and aesthetic outcomes (including patient-reported satisfaction where available). Finally, the key conclusions of each study were documented to capture insights regarding the comparative effectiveness of fully guided versus free-hand approaches.

### 2.5. Collating, Summarizing, and Reporting Results

Extracted data were synthesized into results tables to facilitate a descriptive analysis of the key findings. Given the scoping nature of this review, a meta-analysis was not performed. Instead, a qualitative synthesis of the principal outcomes was conducted to explore and evaluate the comparative effectiveness of fully guided versus conventional free-hand orthognathic surgery techniques. This synthesis focused on delineating differences in surgical accuracy, operative efficiency, functional and aesthetic outcomes, and complication profiles, thereby mapping the current state of evidence and identifying gaps to guide future research in this evolving field.

## 3. Results

**Figure 1:**
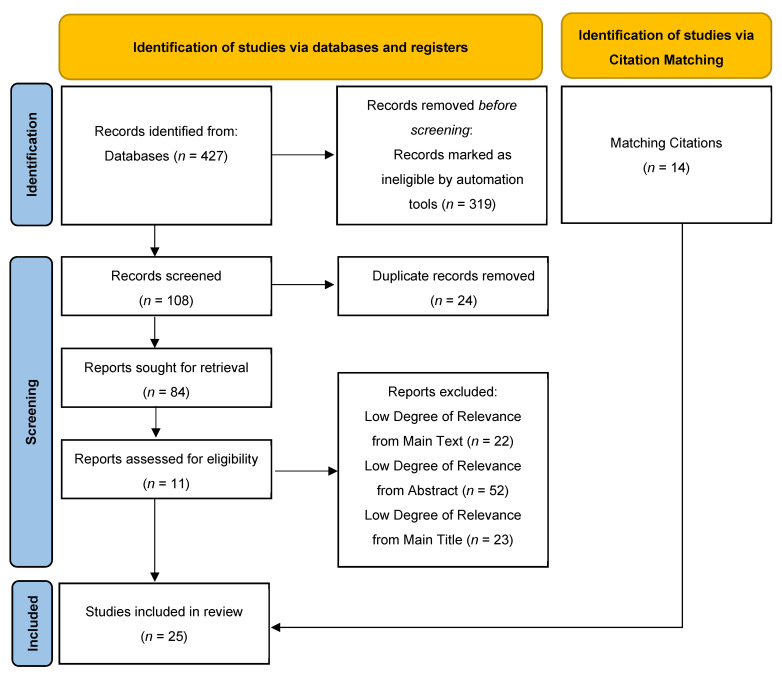
PRISMA flow diagram.

The PRISMA flow diagram (Figure 1) outlines the review selection and exclusion process. Initially, a total of 427 records were retrieved from the aforementioned databases (PubMed and MEDLINE, *n* = 208; Scopus, *n* = 183; Cochrane and Embase, *n* = 36). Automated screening excluded 319 records, leaving 108 records for further consideration. Of these, 24 duplicate records were removed manually. Subsequently, 73 studies were excluded based on ineligible study design, as determined through title and abstract screening. After a full-text review of the remaining 11 articles, all the studies met the inclusion criteria and, thus, were included in the review. From the review of references for identification of any additional relevant articles, 14 additional studies were included. Thus, a total of 25 studies were included in this scoping review.

Included studies were published between 2013 and 2022 (Table 2). Across multiple studies, fully guided surgery demonstrated superior accuracy, with intraoperative navigation achieving length and angular deviations between 1.34 mm and 2.4 mm and 2.29° and 3.51°, respectively, particularly in vertical positioning. Digital planning methods such as virtual surgical planning (VSP) and computer-aided design/computer-aided manufacturing (CAD/CAM) allowed for precise skeletal realignment, reducing intraoperative adjustments and improving postoperative symmetry.

When it comes to operative efficiency, computer-assisted approaches significantly reduced surgical duration compared to free-hand methods, ranging from 34 min to 1.7 h. The use of pre-bent osteosynthesis plates and patient-specific guides streamlined workflows, with studies reporting a reduction in total operative time. Additionally, improved preoperative simulations facilitated faster execution and reduced intraoperative revisions.

Postoperative outcomes indicate that patients undergoing fully guided surgery experienced shorter hospital stays and fewer complications. The enhanced precision of digital planning resulted in higher patient satisfaction scores, with improved functional and aesthetic outcomes compared to free-hand techniques.

The comparative effectiveness of both approaches is outlined in Table 2, providing a detailed analysis of surgical accuracy, efficiency, postoperative results, and complication rates across various studies.

## 4. Discussion

Orthognathic surgery demands precise skeletal repositioning to optimize both functional outcomes and facial aesthetics. A considerable body of literature has addressed the comparative merits of fully guided, computer-assisted approaches and conventional free-hand techniques. The former utilizes VSP, three-dimensional (3D) modeling, CAD/CAM, and rapid prototyping to generate patient-specific cutting guides, pre-bent osteosynthesis plates, and splints, thereby creating a workflow that facilitates accurate preoperative simulation, replicable intraoperative execution, and improved predictability of outcomes. Conversely, traditional free-hand methods rely on conventional cephalometric analyses, stone model surgery, and manual fabrication processes that are inherently dependent on the surgeon’s experience and can be prone to inter- and intra-operator variability.

Fully guided approaches have revolutionized preoperative planning by incorporating three-dimensional imaging, virtual surgical planning (VSP), CAD/CAM, and rapid prototyping to generate patient-specific guides, cutting templates, and splints. The digital methods enable surgeons to simulate skeletal movements with submillimetric precision, effectively “translating” a meticulously designed virtual plan into the operating suite [9,13,30]. On the contrary, conventional free-hand techniques rely on two-dimensional cephalometric tracings, stone model surgery, and manual splint fabrication—a process that inherently involves multiple laboratory steps [23,24]. Although such traditional methods can achieve acceptable outcomes when performed by experienced surgeons, they are more susceptible to errors due to human handling and the limitations of 2D data when representing complex 3D anatomy [12,25].

Studies consistently report that the fully guided approach yields superior accuracy and reproducibility. For instance, Zhang L et al., 2016, found that digitally planned osteotomies produced mean linear deviations of approximately 1.34 mm and angular deviations near 2.29°, results that are less variable than those obtained with free-hand surgery [14]. Similarly, multiple articles [11,15,20] indicate that guided methods offer improved replication of the preoperative design, with enhanced control over fibular segment positioning and mandibular arc restoration. Even though De Maesschalck T et al., 2017, acknowledged that highly experienced free-hand surgeons can achieve comparable outcomes, the digital approach reduces inter-operator variability and provides standardized, measurable endpoints, thus fostering consistency across a wider range of clinical scenarios [15].

A prominent advantage of fully guided techniques is the reduction in operative time and associated ischemia. The utilization of patient-specific guides and prebent plates has been documented in numerous reports [9,10,17,18] to show a significant reduction in surgical durations. The guided approach eliminates time-consuming intraoperative adjustments, such as manual plate bending and repetitive trial fragment positioning, which streamlines the operating room workflow. This results in measurable reductions in both overall surgery time and flap ischemia periods. Furthermore, research conducted during the preoperative phase [24,27,29] has demonstrated that digital planning significantly reduces laboratory work, thereby reducing the overall planning time and alleviating the stress on resident training schedules.

The improved aesthetic and functional outcomes are a clear indication of the precision that computer-assisted planning provides. It is well-established that fully guided techniques result in superior mandibular symmetry, improved occlusal relationships, and improved condyle positioning, all of which contribute to satisfactory masticatory function and facial aesthetics [13,21,31,32]. The guided method is frequently preferred by objective measures such as soft tissue prediction and landmark accuracy [22,25] in contexts where subjective aesthetic preferences differ, even though certain free-hand cases exhibit high patient satisfaction [21]. These enhancements are indispensable in complex orthognathic cases, as even minor discrepancies can lead to long-term functional impairment or facial asymmetry.

Although fully guided techniques require a higher upfront investment, due to the cost of specialized equipment, software, and custom fabrication [16,26,30] several studies argue that these initial expenses are offset by subsequent savings. Particularly in high-volume centers, an overall cost-effective profile is facilitated by reduced operative time, shorter ischemia durations, and a decrease in corrective revision surgeries [17,26]. The economic analysis emphasizes that digital planning can result in substantial annual savings and improved departmental efficiency when indirect costs (such as operating room time and surgeon labor) are considered.

A further consideration is the impact on surgical training and the flexibility of the technique. Fully guided approaches can shorten the learning curve for less experienced surgeons by providing a clear, reproducible digital plan [11,27]. However, concerns have been raised regarding a potential overreliance on digital workflows at the expense of traditional tactile skills—a particularly important factor when considering unusual or unanticipated intraoperative scenarios [12,18]. In contrast, the free-hand approach offers greater adaptability during surgery, enabling real-time adjustments in response to intraoperative findings, although this flexibility may come at the cost of increased variability and prolonged operative duration [9,19].

In maxillary repositioning and bimaxillary surgeries, both digital and conventional techniques produce clinically acceptable outcomes. For instance, Kwon TG et al., 2014, and Ritto FG et al., 2018 [23,28] demonstrated that the error margins for maxillary repositioning using digital methods are within 1–2 mm, with some studies reporting better alignment and occlusion. Additionally, Schwartz HC, 2014, and Van Hemelen G et al., 2015 [24,25] further support that the 3D guided approach enhances soft tissue prediction and overall symmetry, factors that are critical in achieving optimal facial balance in bimaxillary orthognathic surgeries.

Prospectively, the integration of emerging digital technologies, such as direct digital intraoral scanning and in-house desktop 3D printing, promises to further streamline the fully guided approach, potentially reducing both cost and planning time even further [26,30]. Prospective, large-scale studies are warranted to validate these early findings and to determine the long-term impacts on functional outcomes, patient satisfaction, and training paradigms.

Despite the promising outcomes associated with fully guided orthognathic surgery, several limitations deserve consideration. In particular, the heterogeneity in study designs, the variability of patient populations, and the use of disparate outcome measures across the literature pose significant challenges to drawing generalized conclusions and directly comparing digital and free-hand techniques. In addition, the practical implications of implementing fully guided approaches extend beyond clinical accuracy: the high initial investment for digital tools, software, and custom fabrication, coupled with the need for specialized surgeon training, may restrict broader adoption—especially in resource-constrained settings. These fiscal and infrastructural constraints underscore the necessity for standardized protocols and cost-effective strategies, as well as enhanced training curricula that integrate both digital and traditional surgical skills. Future investigations should prioritize robust, multicenter studies that not only harmonize methodological approaches but also explore scalable implementation models to bridge the gap between technological advancements and real-world surgical practice.

## 5. Conclusions

Fully guided techniques in orthognathic surgery substantially improve surgical precision, reproducibility, and workflow efficiency in comparison to traditional free-hand methods. The consistent replication of preoperative simulations and submillimetric accuracy is made possible by digital planning and patient-specific guides, which leads to enhanced functional and aesthetic outcomes with a reduced operative time. The overall benefits of these computer-assisted approaches, which include reduced variability and potential cost-effectiveness in high-volume settings, emphasize the transformative potential, despite the initial investment and potential loss of intraoperative flexibility. The validation of long-term results and the investigation of hybrid strategies that integrate the adaptability of traditional techniques with digital precision should be the primary focus of future research.

## Figures and Tables

**Table 1:** Population, intervention, comparator, outcome table (PICO).

P (Population)	Adult (≥18 years) patients undergoing orthognathic surgery
I (Intervention)	Fully guided (digital, computer-assisted) surgical approaches, incorporating virtual surgical planning, CAD/CAM, and 3D imaging
C (Comparator)	Conventional free-hand techniques
O (Outcome)	Surgical accuracy, operative efficiency, functional and aesthetic outcomes, and reducing complication rates

**Table 2:** Table of results from the included studies.

Author, Year	Study Design; Sample Size	Patient Age	Procedure Type	Intervention Type	Pre/Intraoperative Planning Protocols	Surgical Accuracy Metrics	Operative Parameters	Complication Rates	Functional; Aesthetic Outcomes	Key Conclusions
Hanasono MM et al., 2013 [9]	Case-Control study; 38	51.0 ± 17.4 years	Mandibular reconstruction	Fully guided (CAD/RPM) vs. free-hand	VSP with custom cutting guides and prebent hardware contrasted with free-hand resection relying on intraoperative judgment	Lower aggregate landmark deviations; improved symmetry	Significant operative time savings, particularly in single free-flap cases	N/A	Improved anatomical alignment and facial symmetry	Fully guided methods yield enhanced precision and efficiency.
Ma H et al., 2021 [10]	Retrospective study; 118	55.8 ± 18 years	Orthognathic and Maxillofacial Reconstructive Surgery	Computer-assisted surgery (CAS) vs. free-hand	Preoperative 3D planning with custom templates versus conventional free-hand planning	Postoperative positioning similar; quantitative metrics not detailed	Reduced overall operative, ischemia, bleeding, hospital and ICU stays	Lower early complication rates in the guided group	Comparable occlusion and function; slight differences in patient-centered measures	CAS reduces operative time and resource utilization, while long-term outcomes are largely similar.
Liu YF et al., 2014 [11]	Retrospective study; 15	39.8 years	Mandibular reconstruction	Fully guided (using custom templates) vs. free-hand	3D preoperative design with cutting guides versus surgeon’s intraoperative judgment	Mean length deviation ~2.40 mm; angular deviation ~3.51°	Reduction in operative time by nearly 2 h	Early complications fewer in the guided group (reported as 1 in 15 vs. 2 in 7 cases)	Slight functional improvements; both methods restored acceptable function and aesthetics	The guided approach offers enhanced precision and shorter operative duration than free-hand methods.
Ciocca L et al., 2015 [12]	Prospective study; 10	N/A	Maxillofacial Surgery	Fully guided (CAD/CAM) vs. free-hand (pre-plating)	Patient-specific cutting guides produced via CAD, versus reliance on intraoperative free-hand adjustments	Trends toward better lateral and arch conformity; vertical differences not statistically significant	Operative time details not specified	N/A	Acceptable restoration; slightly better reproducibility with guided approach	Fully guided techniques improve reproducibility though experienced surgeons can achieve acceptable free-hand results.
Weitz J et al., 2016 [13]	Retrospective study; 50	56 years (SD 13) vs. 55 years (SD 16)	Mandibular reconstruction	Fully guided using VSP with stereolithographic models and cutting guides vs. free-hand	Detailed virtual planning with patient-specific cutting guides; intraoperative application of the digital plan	Smaller postoperative deviations in mandibular angles	Approximate reduction in operating time by 34 min; improved bone consolidation	Similar early complication profiles	Improved occlusion, facial symmetry, and long-term bony union	Digital planning enhances predictability and efficiency, especially in complex reconstructions.
Zhang L et al., 2016 [14]	Retrospective study; 22	35.5 years	Mandibular reconstruction	Fully guided (CAD/CAM-assisted) vs. conventional free-hand	Virtual surgical planning with custom cutting guides compared to manual intraoperative adjustments	Mean length deviation ~1.34 mm; angular deviation ~2.29°	Reduced ischemia time (~52.5 min in guided vs. 94.2 min in free-hand)	N/A	Superior occlusion and bone-to-bone contact; enhanced symmetry	Fully guided methods yield lower deviations and decreased operative time compared to free-hand methods.
De Maesschalck T et al., 2017 [15]	Retrospective study; 18	65.8 years vs. 55.9 years	Mandibular reconstruction	Fully guided (CAS) vs. free-hand	Use of 3D virtual planning with patient-specific instruments versus conventional free-hand techniques	Deviations reported in ranges: length deviations 1.3–2.4 mm, angular deviations 2.29°–3.51°	Overall outcomes comparable; influenced by operator experience and learning curve	Comparable rates reported	Acceptable morphological outcomes with minor advantages for guided procedures	CAS may offer benefits in consistency, particularly for less experienced surgeons, though free-hand can be effective with expertise.
Sieira Gil R et al., 2015 [16]	Prospective study; 20	47 years (SD 14) vs. 64 years (SD 13)	Mandibular reconstruction	Fully guided using CAD and RPM vs. free-hand	CAD-based preoperative planning producing patient-specific cutting guides and precontoured plates compared to manual plate bending	Enhanced replication of mandibular contours observed	Reduced operating time—savings ranging from 42 min up to 1.7 h reported	Fewer early complications reported	Improved occlusion and facial aesthetics due to precise bone contact	Fully guided approaches streamline intraoperative procedures despite higher preoperative costs.
Zweifel DF et al., 2015 [17]	Prospective Study; 9	65.9 years vs. 57.5 years	Head and neck free-flap reconstructions (mandibular focus)	Fully guided (VSP and 3D planning) vs. free-hand	Use of digital planning to generate patient-specific templates, allowing precise flap sculpting versus conventional adjustments	Not directly measured	Operating time reduced by 60–102 min; cost-saving estimated at US $47.50 per minute	N/A	Indirectly supports better functional outcomes through precision	The improved operative efficiency and time savings of the fully guided approach result in significant cost-effectiveness.
Tarsitano A et al., 2016 [18]	Prospective study; 4	N/A	Mandibular reconstruction	Fully guided (CAD/CAM) vs. free-hand	Virtual preoperative planning with patient-specific cutting guides and prebent plates versus free-hand manual plate bending	Improved replication of the native mandibular contour; better lateral accuracy observed	Fat reduction in fibular segment preparation time from 26 min (free-hand) to 10 min (guided), contributing to overall time reduction	N/A	Improved occlusal function and aesthetic outcomes reported	The fully guided approach markedly reduces operative time while enhancing reproducibility and accuracy.
Wang YY et al., 2016 [19]	Retrospective Study; 56	52 years	Mandibular reconstruction (free fibula flap)	Fully guided vs. conventional free-hand	Preoperative digital planning generating patient-specific guides compared with conventional, surgeon-dependent methods	Enhanced anatomical accuracy with lower deviation values demonstrated	Notable decrease in ischemia time (e.g., guided group at ≈70 min) and overall surgical time	Fewer alignment-related complications reported	Improved bone consolidation and occlusion outcomes	Fully guided techniques better replicate the preoperative plan with increased efficiency and functional outcomes.
Culié D et al., 2016 [20]	Retrospective study; 29	64.8 ± 8.9 years vs. 60.6 ± 10.9 years	Mandibular reconstruction	Fully guided (CAD/CAM) vs. conventional free-hand	Digital design of cutting guides ensuring precise bone segmentation vs. free-hand intraoperative adjustments	Trend toward superior lateral and vertical alignment of fibular segments	Accelerated osteotomies due to guided cutting, reducing total operative time	N/A	Improved restoration of the mandibular arch with better symmetry	Fully guided reconstruction offers more reliable contour restoration relative to conventional methods.
Bouchet B et al., 2018 [21]	Monocentric Retrospective study; 25	59.2 years vs. 60.2 years	Mandibular reconstruction	Fully guided (CAD/CAM-assisted) vs. conventional free-hand	Patient-specific cutting guides and precontoured plates developed via CAD versus manual techniques	Not numerically specified, but objective measures (e.g., reduced chin deviation) improved	Operative time details not specified	N/A	Objective measures (e.g., range of motion) favored guided methods, though free-hand cases sometimes reported higher subjective satisfaction	CAD/CAM-assisted techniques improve objective functional parameters, although subjective aesthetic ratings may vary.
Bartier S et al., 2021 [22]	Retrospective study; 33	55.9 ± 12.7 years	Mandibular reconstruction (free fibula flap)	Fully guided (CAD/CAM with VSP and cutting guides) vs. free-hand	Thorough VSP with integration of multiple anatomical checkpoints and fabrication of custom guides versus intraoperative free-hand adjustment	Significantly improved sagittal/coronal symmetry and condyle positioning observed	No significant difference in overall operative time reported	N/A	Superior aesthetic outcomes and functional reproducibility demonstrated	Fully guided reconstruction achieves improved midskeletal symmetry and may enhance overall outcomes.
Kwon TG et al., 2014 [23]	Retrospective study; 42	21.9 ± 3.0 years vs. 23.1 ± 5.2 years	Maxillary (Le Fort I osteotomy)	Fully guided (Virtual Model Surgery, VMS/Digital) vs. conventional free-hand (Analog Model Surgery, AMS)	Integration of 3D dental data and cephalometric analysis with rapid digital model fabrication versus traditional impression-based methods	Discrepancy within 1 mm in 63.2% of VMS cases vs. 26% in AMS cases	Reduced laboratory fabrication time; streamlined digital workflow	N/A	Achieved comparable clinical reliability with improved precision in angular and linear measurements	Digital VMS presents clear workflow advantages without compromising clinical accuracy.
Schwartz HC, 2014 [24]	Retrospective study; 30	28.3 years	Bimaxillary orthognathic surgery	Fully guided (CASS—Computer-Assisted Surgical Simulation) vs. conventional free-hand planning	Extensive computer-assisted preoperative planning with multiple appointments versus traditional manual planning with dental casts	Not applicable—focus mainly on time and resource use	Total doctor time reduced from an average of 865 min to 805 min per case (~60 min saved)	N/A	Enhanced overall efficiency potentially leading to increased surgical throughput	CASS significantly reduces planning time and may free up clinical resources in high-volume centers.
Van Hemelen G et al., 2015 [25]	Randomized Prospective study; 66	19.78 years	Orthognathic surgery	Fully guided (3D computer-aided planning) vs. conventional free-hand (2D planning)	Preoperative 3D digital modeling for soft and hard tissue outcomes versus traditional 2D cephalometric analysis and model fabrication	Statistically significant improvement in soft tissue prediction; hard tissue error differences (<2 mm) acceptable	Operative time not detailed; focus on planning predictability	N/A	Enhanced facial symmetry and better soft tissue outcomes	3D guided planning offers improved predictability for soft tissue outcomes in complex cases.
Resnick CM et al., 2016 [26]	Retrospective study; 43	N/A	Bimaxillary orthognathic surgery	Fully guided (VSP with 3D-printed splints) vs. conventional free-hand	Digital workflow integrating 3D-printed splints vs. traditional plaster model surgery and manual splint fabrication	Not applicable—focus on economic parameters	Estimated cost savings of ~$650–$930 per case, with further financial benefit when splint costs are excluded; annual time savings (~25 working days across 200 cases)	N/A	Clinical outcomes acceptable in both groups, with digital planning enhancing predictability	VSP-based planning is demonstrably time-efficient and cost-effective when considering overall resource utilization.
Wrzosek MK et al., 2016 [27]	Prospective study; 41	N/A	Bimaxillary orthognathic surgery	Fully guided (VSP with 3D-printed splints) vs. conventional free-hand	Office-based digital planning reducing extensive manual laboratory steps compared to traditional model preparation	Improvements in reproducibility noted (exact numbers not provided)	Preoperative planning time reduced by approximately 2.2 h; significant reduction in resident workload	N/A	Maintained or improved occlusal and skeletal accuracy with greater planning efficiency	VSP significantly cuts planning time and labor, ultimately benefiting training and departmental throughput.
Ritto FG et al., 2018 [28]	Retrospective study; 30	N/A	Maxillary repositioning	Fully guided (VSP) vs. conventional (CMS—model surgery-based planning)	Utilization of cone-beam CT data and digital simulation versus traditional dental cast mounting and articulator-based model surgery	Mean linear error approx. 1.20 mm for VSP vs. 1.27 mm for CMS	Not specified in detail; emphasis on improved workflow in digital preoperative planning	N/A	Functional outcomes comparable; additional planning workflow benefits noted	VSP achieves equivalent maxillary accuracy with the added benefit of streamlined preoperative procedures.
Steinhuber T et al., 2018 [29]	Prospective Control Study; 40	24.6 years	Orthognathic surgery (single- and double-jaw)	Fully guided (office-based VSP) vs. conventional free-hand planning	Digital planning performed by experienced technicians minimizing manual laboratory steps versus conventional labor-intensive model preparation	Not reported—primary focus on planning time efficiency	Overall planning time savings: 36 min (single-jaw) and 74 min (double-jaw); surgeon’s direct planning time similar	N/A	Maintained clinical outcomes with improved workflow efficiency	VSP markedly reduces overall planning time, decreasing resident workload and increasing departmental efficiency.
Schneider D et al., 2019 [30]	Randomized Controlled Trial; 21	31.1 years	Orthognathic surgery	Fully guided (VSP with CAD/CAM and 3D printing) vs. conventional free-hand	Advanced digital workflow allowing for rapid modifications and pre-bent plate simulation versus traditional cephalometric tracing and stone model adjustment	Lower angular errors (SNA, SNB, ANB) and improved splint accuracy reported	Reduced intraoperative adjustments; approx. 31% reduction in time for splint-based interventions	N/A	Enhanced functional outcomes and improved facial symmetry	Fully guided VSP offers significant advantages in precision and intraoperative efficiency.
Al-Sabahi ME et al., 2022 [31]	Prospective Randomized Control Trial; 22	41 ± 18.5 years vs. 47.81 ± 13.6 years	Mandibular reconstruction	Fully guided (CAD/CAM-assisted, “COG” group) vs. conventional free-hand (“MB” group)	Digital planning with patient-specific cutting guides and pre-bent plates compared to conventional free-hand reconstruction	Demonstrated improved mandibular contour symmetry with lower angular deviations	Significantly shorter operating and ischemia times reported in the guided group	N/A	Higher patient satisfaction scores (VAS, PSS) and enhanced facial aesthetics	Fully guided techniques result in superior aesthetic symmetry and operative efficiency compared to free-hand methods.
Bao T et al., 2017 [32]	Retrospective study; 35	N/A	Mandibular reconstruction	Fully guided (CAD/CAM) vs. conventional free-hand	Computer-aided 3D modeling to create patient-specific cutting guides and pre-bent titanium plates vs. reliance on intraoperative judgment and manual adjustments	Demonstrated improved precision in osteotomy angles, fibular segment lengths, and positioning	Reported mean ischemia time ~70 min in the guided group vs. 120–180 min in free-hand; overall operative time shorter	N/A	Improved occlusal relationships and facial symmetry; reduced tissue trauma	Fully guided CAD/CAM techniques significantly enhance surgical accuracy, decrease ischemia time, and improve predictability, despite higher upfront costs.
Ritschl LM et al., 2017 [33]	Retrospective study; 30	63.07 ± 8.08 years vs. 61.94 ± 11.64 years	Mandibular reconstruction	Fully guided (CAD/CAM/virtual planning) vs. conventional free-hand	Virtual planning with 3D modeling, patient-specific cutting guides, and pre-bent osteosynthesis plates vs. conventional intraoperative adjustments	Improved replication of native mandibular anatomy with reduced deviations, though functional measures (e.g., mouth opening) were comparable	Trend toward shorter overall operative time, with an average saving of ~35 min observed in the guided group	No statistically significant difference reported	Comparable functional outcomes; improved predictability in complex cases with digital planning	Fully guided techniques offer advantages in replicating native mandibular contours and reducing operative time, particularly in complex cases, although functional outcomes are similar to free-hand methods.

## Supplementary Materials

The following supporting information can be downloaded at: https://www.mdpi.com/article/10.3390/dj13060260/s1, Table S1: Preferred Reporting Items for Systematic reviews and Meta-Analyses extension for Scoping Reviews (PRISMA-ScR) Checklist.
